# Effect of Pneumococcal Conjugate Vaccines on Pneumococcal Meningitis, England and Wales, July 1, 2000–June 30, 2016

**DOI:** 10.3201/eid2509.180747

**Published:** 2019-09

**Authors:** Godwin Oligbu, Sarah Collins, Abdelmajid Djennad, Carmen L. Sheppard, Norman K. Fry, Nick J. Andrews, Ray Borrow, Mary E. Ramsay, Shamez N. Ladhani

**Affiliations:** Public Health England, London, UK (G. Oligbu, S. Collins, A. Djennad, C.L. Sheppard, N.K. Fry, N.J. Andrews, M.E. Ramsay, S.N. Ladhani);; St. George's, University of London, London (G. Oligbu, S.N. Ladhani);; Public Health England Manchester Royal Infirmary, Manchester, UK (R. Borrow)

**Keywords:** pneumococcal meningitis, *Streptococcus pneumoniae*, conjugate vaccines, epidemiology, case-fatality rate, England, Wales, PCV7, PCV13, serotype, incidence, immunization, bacteria, meningitis, serotype 8, case fatality rate, invasive pneumococcal disease

## Abstract

We describe the effects of the 7-valent (PCV7) and 13-valent (PCV13) pneumococcal conjugate vaccines on pneumococcal meningitis in England and Wales during July 1, 2000–June 30, 2016. Overall, 84,473 laboratory-confirmed invasive pneumococcal disease cases, including 4,160 (4.9%) cases with meningitis, occurred. PCV7 implementation in 2006 did not lower overall pneumococcal meningitis incidence because of replacement with non–PCV7-type meningitis incidence. Replacement with PCV13 in 2010, however, led to a 48% reduction in pneumococcal meningitis incidence by 2015–16. The overall case-fatality rate was 17.5%: 10.7% among patients <5 years of age, 17.3% among patients 5–64 years of age, and 31.9% among patients >65 years of age. Serotype 8 was associated with increased odds of death (adjusted odds ratio 2.9, 95% CI 1.8–4.7). In England and Wales, an effect on pneumococcal meningitis was observed only after PCV13 implementation. Further studies are needed to assess pneumococcal meningitis caused by the replacing serotypes.

*Streptococcus pneumoniae* is a major cause of bacterial meningitis across all age groups in the United Kingdom and worldwide (*1*,*2*); the case-fatality rate (CFR) ranges from 10% to 40% (*2*–*4*). Survivors of pneumococcal meningitis are more likely than survivors of other types of bacterial meningitis to have neurologic and other serious long-term sequelae (*5*,*6*); a meta-analysis indicated that 32% of pneumococcal meningitis patients experienced sequelae (*7*). The pathophysiologic mechanisms leading to neurologic damage in patients with bacterial meningitis are complex and multifaceted, involving the secretion of potent bacterial toxins and excessive host immune responses against the invading pneumococci in the cerebrospinal fluid (*8*,*9*).

Before the introduction of the 7-valent pneumococcal conjugate vaccine (PCV7), ≈500 confirmed pneumococcal meningitis cases occurred annually in England and Wales (*2*). The serotypes covered by PCV7 were responsible for 57% of all pneumococcal meningitis cases and 72% of cases in children <2 years of age; the CFR increased with age, from 5% in children to 30% in older adults (*10*).

In September 2006, the United Kingdom introduced PCV7 into the childhood immunization program; children were scheduled to receive the vaccine at 2, 4, and 12 months of age, and a 12-month catch-up program was established for children <2 years of age (*11*). The program was associated with a rapid decline in invasive pneumococcal disease (IPD) caused by PCV7 serotypes, and although some increase in IPD caused by non-PCV7 serotypes was observed, IPD decreased overall by 36% compared with pre-PCV7 levels through direct and indirect protection (*12*). During the first 4 years of the program, a 34% reduction in pneumococcal meningitis incidence was observed in children <5 years of age (*13*). However, this reduction was almost entirely offset by an increase in meningitis cases caused by non-PCV7 serotypes in older children and adults. After PCV7 introduction, pneumococcal meningitis was mainly caused by serotypes 1, 3, 7F, 19A, 22F, and 33F (*14*).

In April 2010, PCV7 was replaced with the 13-valent vaccine (PCV13), which led to a 32% reduction in overall IPD incidence compared with pre-PCV7 levels and a 56% reduction compared with pre-PCV13 levels (*14*). The effect of PCV13 on pneumococcal meningitis has not been assessed in the United Kingdom. Reports of the effects of PCV7 and PCV13 on pneumococcal meningitis in other countries with established pneumococcal immunization programs have been variable; in some countries, significant reductions were reported after PCV7 introduction, and in other countries, no change or a decline was reported only after PCV13 introduction (*15*–*20*). Here, we describe the epidemiology of pneumococcal meningitis in England and Wales over a 16-year period encompassing the introduction of PCV7 and PCV13 into the national immunization program.

## Methods

### Surveillance

Public Health England (PHE; London, England, UK) has legal permission under Regulation 3 of the Health Service (Control of Patient Information) Regulations 2002 (http://www.legislation.gov.uk/uksi/2002/1438/regulation/3/made) to conduct national surveillance of communicable diseases. This regulation also provides PHE permission to access information required to monitor the safety and effectiveness of vaccines.

PHE conducts surveillance for IPD and provides a national reference service for serotyping pneumococcal isolates in England and Wales (*12*). Staff of the National Health Service laboratories electronically report invasive bacterial infections to PHE by using the Second Generation Surveillance System, which replaced LabBase2 in 2014, and routinely submit all invasive pneumococcal isolates to the PHE national reference laboratory for confirmation and serotyping (*12*). PHE staff actively follow up on reported cases when they do not receive an accompanying isolate. Case ascertainment has remained consistently high, especially for meningitis cases; >90% of invasive pneumococcal isolates are submitted to PHE for serotyping (*12*). Starting in September 2006, IPD surveillance was enhanced by the collection of 1-page surveillance questionnaires completed by the patient’s general practitioner; questionnaires asked for information on patients’ vaccination histories, underlying medical conditions, and outcomes.

### Data Analysis

We exported anonymized (mainly descriptive) data to Stata v.11.0 (https://www.stata.com) for analysis. We included laboratory-confirmed cases of pneumococcal meningitis diagnosed during July 1, 2000–June 30, 2016 (16 epidemiologic years). We defined meningitis as identification of *S. pneumoniae* in cerebrospinal fluid or blood cultures of patients with a clinical diagnosis of meningitis, as designated on their electronic report or sample submission form sent to PHE. Excluding clinically diagnosed cases in which *S. pneumoniae* was not confirmed in the cerebrospinal fluid (25%–35% of all pneumococcal meningitis cases annually) reduced the total number of meningitis cases available for analysis without affecting the observed trends over time. We identified fatal cases and dates of death through the patient demographic service and calculated the 30-day CFR.

We classified cases into 4 groups by serotype: PCV7 (serotypes 4, 6B, 9V, 14, 18C, 19F, 23F), additional PCV13 (serotypes 1, 3, 5, 6A, 7F, 19A), non-PCV13, and unknown (typically resulting from lack of referral or unsuccessful recovery from culture after sample transport). We analyzed cases as a whole and by patient age group (<5, 5–64, >65 years). We filled in missing age and serotype by assuming that age and serotype distribution were the same on reports with missing information as on reports for which these parameters were known. We obtained age-specific population denominators from the Office for National Statistics (www.statistics.gov.uk) and compared the adjusted annual incidence rates for pneumococcal meningitis in epidemiologic year 2015–16 with rates for the pre-PCV7 (July 1, 2000–June 30, 2006) and pre-PCV13 (July 1, 2008–June 30, 2010) periods by age and serotype group, assuming a Poisson distribution.

For cases diagnosed during the PCV13 period (July 1, 2011–June 30, 2016), we used multivariable logistic regression to calculate the odds of meningitis (vs. nonmeningitis) for individual serotypes (vs. all other serotypes) after adjusting for age group and surveillance year. We also used multivariable logistic regression to calculate the odds of death by clinical presentation (meningitis vs. nonmeningitis) after adjusting for age group and surveillance year. For meningitis cases, we used multivariable logistic regression to assess the odds of death by age group, serotype group, individual serotype (vs. all other serotypes), and surveillance year after adjusting for age group and surveillance year. Because of multiple comparisons, we considered p<0.01 to be significant. We estimated the cases prevented according to age group by determining the difference between the expected (average corrected number of cases in absence of vaccination) and observed numbers of cases after the introduction of each vaccine.

## Results

During the 16-year surveillance period, 84,473 laboratory-confirmed IPD cases occurred across all age groups; 4,160 (4.9%) cases were meningitis and 80,313 (95.1%) were nonmeningitis. Of the 4,108 meningitis cases with age reported, 1,611 (39.2%) were in children <5 years of age, 1,729 (42.1%) in persons 5–64 years of age, and 768 (18.7%) in adults >65 years of age. Of the 79,620 nonmeningitis cases with age reported, 8,324 (10.5%) were in children <5 years of age, 32,297 (40.6%) in persons 5–64 years of age, and 38,999 (48.9%) in adults >65 years of age.

**Figure 1:**
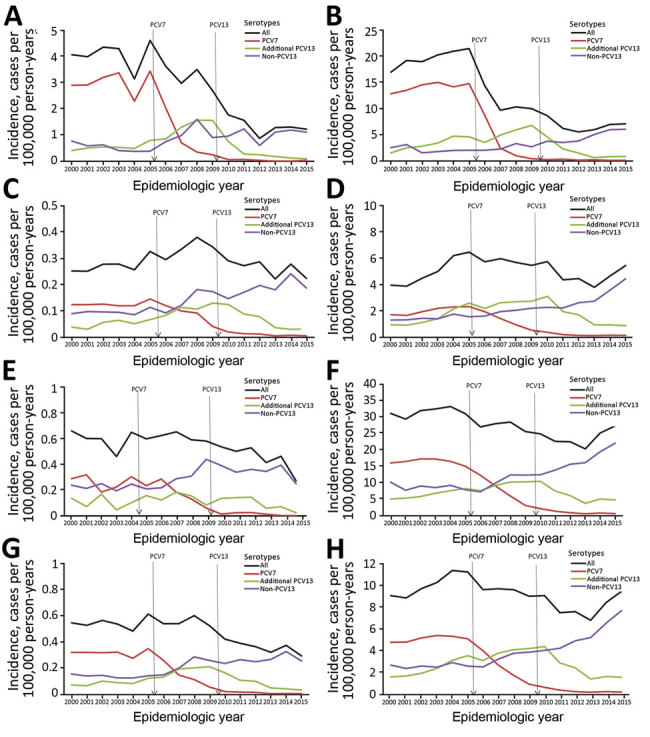
Corrected trends in incidence of pneumococcal meningitis and nonmeningitis cases by *Streptococcus pneumoniae* serotype, age group, and epidemiologic year, England and Wales, July 1, 2000–June 30, 2016. A–H) Meningitis (A, C, E, G) and nonmeningitis (B, D, F, H) cases in patients <5 years of age (A, B); patients 5–64 years of age (C, D); patients >65 years of age (E, F); and patients of all ages (G, H). The raw numbers of cases for each year were corrected for missing serotype and age with the assumption that cases with missing data for age, serotype, or both had the same age and serotype distribution as those cases for which this information was known; cases were also corrected for annual changes in population denominators in each age group (*13*).The vertical lines denote the introduction of PCV7 and PCV13 into the national childhood immunization program. PCV7 refers to serotypes 4, 6B, 9V, 14, 18C, 19F, and 23F, and additional PCV13 refers to serotypes 1, 3, 5, 6A, 7F, and 19A. Non-PCV13 refers to all other serotypes. PCV7, 7-valent pneumococcal conjugate vaccine; PCV13, 13-valent pneumococcal conjugate vaccine.

**Figure 2:**
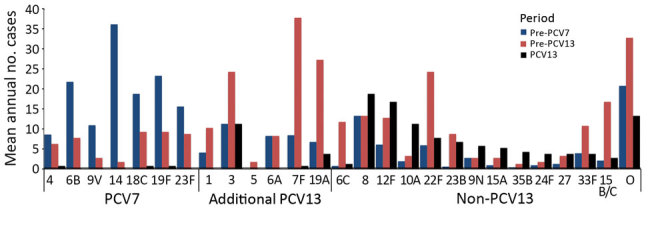
Mean annual number of pneumococcal meningitis cases among patients of all ages by *Streptococcus pneumoniae* serotype and period, England and Wales, July 1, 2000–June 30, 2016. The pre-PCV7 period refers to July 1, 2000–June 30, 2006, pre-PCV13 period July 1, 2008–June 30, 2010, and PCV13 period July 1, 2011–June 30, 2016. PCV7, 7-valent pneumococcal conjugate vaccine; PCV13, 13-valent pneumococcal conjugate vaccine. For cases diagnosed during the PCV13 period (July 1, 2011–June 30, 2016), we used multivariable logistic regression to calculate the odds of meningitis vs. nonmeningitis. O, other serotypes.

Before PCV7 introduction, the mean annual incidence of pneumococcal meningitis was 0.55 cases/100,000 person-years (Table 1). The childhood PCV7 program had no effect on the overall annual incidence of pneumococcal meningitis (pre-PCV13 period 0.56 cases/100,000 person-years) because the decline in PCV7-type meningitis was offset by substantial increases in cases caused by other serotypes (Table 1; Figure 1). PCV7 replacement with PCV13 in April 2010, however, led to a 48% (95% CI 38%–62%) reduction in pneumococcal meningitis incidence by 2015–16. During the PCV13 period, meningitis cases caused by PCV7 and PCV13 serotypes continued to decline, and cases associated with non-PCV13 serotypes remained static. These findings are in contrast with those regarding nonmeningitis IPD cases, in which reductions were observed across all age groups but were offset by increases in cases caused by nonvaccine serotypes after the introduction of PCV7 and PCV13 (Table 1). The serotypes responsible for meningitis varied among the pre-PCV7, pre-PCV13, and PCV13 periods (Figure 2).

**Table 1:** Cases, incidence, and age-adjusted IRRs for pneumococcal meningitis and nonmeningitis cases by age group, serotype group, and period, England and Wales, July 1, 2000–June 30, 2016*

Case type, age group, and serotype group	July 1, 2015–June 30, 2016			Pre-PCV13 period, July 1, 2008–June 30, 2010				Pre-PCV7 period, July 1, 2000–June 30, 2006		
	No. corrected (raw) cases†	Incidence, cases/ 100,000 person-years		No. corrected (raw) cases†	Incidence, cases/ 100,000 person-years	IRR (95% CI) vs. 2015–16		No. corrected (raw) cases†	Incidence, cases/ 100,000 person-years	IRR (95% CI) vs. 2015–16
Meningitis										
<5 y										
All serotypes	41 (44)	1.22		105 (104)	3.10	0.39 (0.25–0.63)		138 (122)	4.08	0.30 (0.19–0.46)
PCV7	1 (1)	0.03		10 (10)	0.29	0.10 (0.01–1.84)		102 (76)	3.02	0.01 (0–0.16)
Additional PCV13	3 (3)	0.09		53 (51)	1.56	0.06 (0.01–0.29)		18 (14)	0.54	0.16 (0.03–0.85)
Non-PCV13	37 (38)	1.10		42 (41)	1.25	0.89 (0.51–1.56)		18 (13)	0.52	2.12 (1.22–3.75)
5–64 y										
All serotypes	96 (98)	0.22		155 (155)	0.36	0.62 (0.44–0.86)		117 (110)	0.27	0.82 (0.60–1.11)
PCV7	2 (2)	0.00		28 (27)	0.07	0.07 (0.01–0.56)		54 (39)	0.13	0.04 (0.01–0.29)
Additional PCV13	14 (13)	0.03		51 (47)	0.12	0.27 (0.11–0.62)		22 (16)	0.05	0.62 (0.26–1.39)
Non-PCV13	80 (77)	0.19		76 (71)	0.18	1.06 (0.71–1.58)		41 (29)	0.10	1.95 (1.31–2.86)
>65 y										
All serotypes	24 (28)	0.27		52 (52)	0.59	0.46 (0.25–0.85)		53 (49)	0.60	0.45 (0.26–0.80)
PCV7	0 (0)	0		8 (8)	0.09	0 (0–0.52)		23 (17)	0.26	0 (0–0.17)
Additional PCV13	2 (2)	0.02		11 (10)	0.12	0.19 (0.02–1.57)		10 (7)	0.11	0.20 (0.02–1.55)
Non-PCV13	22 (22)	0.25		33 (32)	0.37	0.67 (0.33–1.34)		20 (15)	0.22	1.11 (0.56–2.18)
All ages										
All serotypes	162 (170)	0.29		311 (310)	0.56	0.51 (0.40–0.66)		301 (288)	0.55	0.52 (0.41–0.65)
PCV7	3 (3)	0.01		46 (44)	0.08	0.07 (0.01–0.35)		176 (133)	0.32	0.02 (0–0.09)
Additional PCV13	18 (18)	0.03		114 (108)	0.21	0.16 (0.08–0.32)		49 (38)	0.09	0.36 (0.18–0.72)
Non-PCV13	141 (137)	0.25		151 (143)	0.27	0.92 (0.69–1.25)		77 (58)	0.14	1.77 (1.33–2.36)
Nonmeningitis										
<5 y										
All serotypes	241 (257)	7.12		345 (341)	10.19	0.70 (0.57–0.86)		666 (592)	19.67	0.36 (0.30–0.44)
PCV7	5 (5)	0.15		25 (23)	0.74	0.21 (0.05–0.79)		479 (310)	14.17	0.01 (0–0.04)
Additional PCV13	30 (29)	0.89		217 (199)	6.40	0.14 (0.08–0.24)		112 (73)	3.30	0.27 (0.15–0.46)
Non-PCV13	206 (198)	6.08		103 (95)	3.05	2.00 (1.50–2.66)		75 (49)	2.20	2.76 (2.11–3.57)
5–64 y										
All serotypes	2,333 (2,387)	5.44		2,385 (2,377)	5.56	0.98 (0.91–1.05)		2,141 (2,028)	4.99	1.09 (1.01–1.16)
PCV7	57 (55)	0.13		313 (274)	0.73	0.18 (0.12–0.28)		865 (465)	2.02	0.07 (0.04–0.10)
Additional PCV13	375 (359)	0.87		1,163 (1,022)	2.71	0.32 (0.27–0.38)		651 (366)	1.52	0.57 (0.46–0.64)
Non-PCV13	1,901 (1,820)	4.43		909 (799)	2.12	2.09 (1.90–2.30)		625 (355)	1.46	3.04 (2.74–3.30)
>65 y										
All serotypes	2,442 (2,818)	27.29		2,417 (2,391)	27.01	1.01 (0.94–1.08)		2,820 (2,601)	31.51	0.87 (0.82–0.92)
PCV7	49 (53)	0.55		402 (351)	4.49	0.12 (0.08–0.18)		1,466 (775)	16.39	0.03 (0.02–0.05)
Additional PCV13	422 (455)	4.71		913 (803)	10.20	0.46 (0.40–0.53)		574 (318)	6.42	0.73 (0.61–0.82)
Non-PCV13	1,971 (2,127)	22.03		1,103 (970)	12.32	1.79 (1.63–1.95)		779 (411)	8.70	2.53 (2.34–2.77)
All ages										
All serotypes	5,217 (5,467)	9.44		5,135 (5,116)	9.30	0.98 (0.93–1.02)		5,563 (5,321)	10.07	0.89 (0.85–0.93)
PCV7	115 (113)	0.21		735 (648)	1.33	0.15 (0.11–0.20)		2,813 (1,571)	5.09	0.04 (0.03–0.05)
Additional PCV13	862 (844)	1.56		2,290 (2,026)	4.15	0.36 (0.33–0.41)		1,311 (767)	2.37	0.60 (0.53–0.66)
Non-PCV13	4,239 (4,148)	7.67		2,109 (1,866)	3.82	1.92 (1.80–2.05)		1,439 (804)	2.61	2.75 (2.59–2.92)

*PCV7 refers to serotypes 4, 6B, 9V, 14, 18C, 19F, and 23F, and additional PCV13 refers to serotypes 1, 3, 5, 6A, 7F, and 19A. Non-PCV13 refers to all other serotypes. IRR, incident rate ratio; PCV7, 7-valent pneumococcal conjugate vaccine; PCV13, 13-valent pneumococcal conjugate vaccine. †Raw numbers of cases for each year were corrected for missing serotype and age with the assumption that cases with missing data for age, serotype, or both had the same age and serotype distributions as those cases in which this information was known—the number of extra cases were then added to the raw numbers in each category; cases were also corrected for annual changes in population denominators in each age group (*13*).

**Figure 3:**
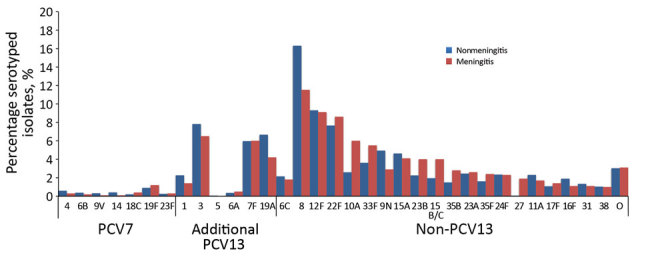
Contribution of individual *Streptococcus pneumoniae* serotypes to pneumococcal meningitis and nonmeningitis cases for all age groups after PCV13 introduction, England and Wales, July 1, 2011–June 30, 2016. We included the non-PCV13 serotypes that involved >10 cases. PCV7, 7-valent pneumococcal conjugate vaccine; PCV13, 13-valent pneumococcal conjugate vaccine. O, other serotypes.

Although meningitis cases were ≈20 times less common than nonmeningitis cases during the 16-year period, the contribution of individual serotypes to these 2 clinical presentations was similar (Figure 3). During the PCV13 period, after adjusting for age and year of diagnosis, odds of causing meningitis were higher for only serotypes 10A, 22F, 23B, and 35B and lower for serotypes 1, 8, and 19A (Table 2).

**Table 2:** Association between *Streptococcus pneumoniae* serotype and meningitis (vs. nonmeningitis) by serotype ranking, England and Wales, July 1, 2011–June 30, 1016*

Serotype rank	Serotype	Total cases	Nonmeningitis cases, n = 20,800, no. (%)	Meningitis cases, n = 920, no. (%)	aOR (95% CI)	p value†
1	8	3,285	3,184 (15.3)	101 (11.0)	0.69 (0.56–0.86)	<0.001
2	12F	1,809	1,729 (8.3)	80 (8.7)	0.99 (0.78–1.27)	0.961
3	22F	1,725	1,640 (7.9)	85 (9.2)	1.39 (1.1–1.76)	0.006
4	3	1,598	1,542 (7.4)	56 (6.1)	0.99 (0.74–1.31)	0.924
5	7F	1,542	1,482 (7.1)	60 (6.5)	0.73 (0.56–0.96)	0.026
6	19A	1,485	1,446 (7.0)	39 (4.2)	0.62 (0.45–0.86)	0.005
7	15A	1,024	986 (4.7)	38 (4.1)	1.09 (0.78–1.54)	0.603
8	9N	954	928 (4.5)	26 (2.8)	0.77 (0.51–1.15)	0.2
9	33F	827	774 (3.7)	53 (5.8)	1.34 (0.99–1.81)	0.057
10	1	602	588 (2.8)	14 (1.5)	0.33 (0.19–0.56)	<0.001
11	10A	566	511 (2.5)	55 (6.0)	2.06 (1.52–2.79)	<0.001
12	23A	548	523 (2.5)	25 (2.7)	1.51 (1–2.29)	0.052
13	24F	548	528 (2.5)	20 (2.2)	0.73 (0.46–1.16)	0.187
14	6C	514	497 (2.4)	17 (1.8)	0.95 (0.58–1.57)	0.852
15	11A	512	498 (2.4)	14 (1.5)	0.71 (0.41–1.23)	0.221
16	23B	499	462 (2.2)	37 (4.0)	1.73 (1.21–2.48)	0.003
17	15B/C	453	416 (2.0)	37 (4.0)	1.31 (0.91–1.89)	0.146
18	16F	418	408 (2.0)	10 (1.1)	0.77 (0.41–1.45)	0.415
19	35F	371	349 (1.7)	22 (2.4)	1.47 (0.93–2.32)	0.096
20	35B	334	308 (1.5)	26 (2.8)	2.12 (1.38–3.25)	<0.001
21	31	282	273 (1.3)	9 (1.0)	1.14 (0.58–2.25)	0.703
22	20	240	235 (1.1)	5 (0.5)	0.46 (0.19–1.12)	0.086
23	38	239	229 (1.1)	10 (1.1)	0.79 (0.41–1.54)	0.49
24	17F	235	221 (1.1)	14 (1.5)	1.71 (0.98–2.98)	0.058
25	19F	183	171 (0.8)	12 (1.3)	1.24 (0.68–2.28)	0.483
26	4	135	132 (0.6)	3 (0.3)	0.4 (0.13–1.28)	0.123
27	14	94	93 (0.4)	1 (0.1)	0.26 (0.04–1.87)	0.18
28	6A	93	88 (0.4)	5 (0.5)	1.34 (0.52–3.45)	0.538
29	6B	90	88 (0.4)	2 (0.2)	0.54 (0.13–0.13)	0.397
30	9V	81	80 (0.4)	1 (0.1)	0.31 (0.04–2.25)	0.245
31	23F	60	57 (0.3)	3 (0.3)	1.24 (0.38–4.08)	0.722
32	18C	55	51 (0.2)	4 (0.4)	1.23 (0.43–3.52)	0.701
33	21	51	47 (0.2)	4 (0.4)	0.8 (0.28–2.3)	0.683
*Values were adjusted for age and year of diagnosis. Only serotypes responsible for >50 cases of invasive pneumococcal disease were included. Serotypes of decreased odds (decreased aORs with significant p values) are indicated in light gray, and serotypes of increased odds (increased aORs and significant p values) are indicated in dark gray. aOR, adjusted odds ratio. †Because of multiple comparisons, a p value of <0.01 was considered significant.						

### Cases in Patients <5 Years of Age

**Figure 4:**
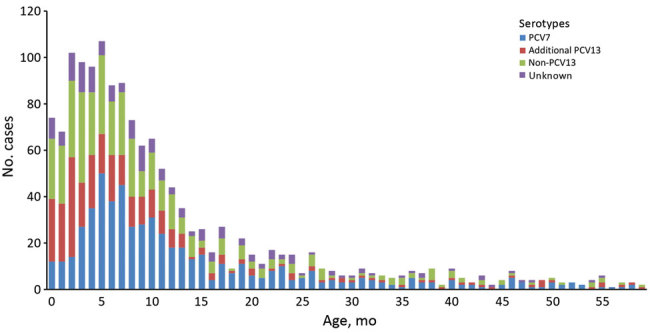
Distribution of pneumococcal meningitis cases in children <5 years of age, by month of age and *Streptococcus pneumoniae* serotype group, England and Wales, July 1, 2000–June 30, 2016. PCV7 refers to serotypes 4, 6B, 9V, 14, 18C, 19F, and 23F, and additional PCV13 refers to serotypes 1, 3, 5, 6A, 7F, and 19A. PCV7, 7-valent pneumococcal conjugate vaccine; PCV13, 13-valent pneumococcal conjugate vaccine.

Pneumococcal meningitis cases in children increased from birth and peaked at 5 months of age, before gradually declining (Figure 4). For patients <5 years of age, PCV7 serotypes contributed to 73.9% (102/138), additional PCV13 to 13.0% (18/138), and non-PCV13 to 13.0% (18/138) of pneumococcal meningitis cases during the pre-PCV7 period (Table 1). After PCV7 introduction, pneumococcal meningitis incidence fell from 4.08 cases/100,000 person-years to 3.10 cases/100,000 person-years in the pre-PCV13 period (Table 1). The rapid decline in PCV7-type meningitis (3.02 cases/100,000 person-years [pre-PCV7] to 0.29 cases/100,000 person-years [pre-PCV13]) was offset by an ≈3-fold increase in PCV13-type disease incidence (0.54 cases/100,000 person-years to 1.56 cases/100,000 person-years), nearly all caused by serotypes 7F (4 cases/year to 26 cases/year), 19A (3 cases/year to 15 cases/year), and 1 (2 cases/year to7 cases/year). In this age group, the incidence of non-PCV13 meningitis also increased from 0.52 cases/100,000 person-years to 1.25 cases/100,000 person-years in the pre-PCV13 period.

**Figure 5:**
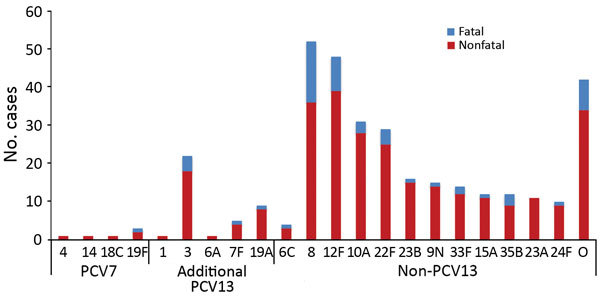
Pneumococcal meningitis cases, by *Streptococcus pneumoniae* serotype and severity, England and Wales, July 1, 2015–June 30, 2016. We included the non-PCV13 serotypes that involved >10 cases. O, other serotypes; PCV7, 7-valent pneumococcal conjugate vaccine; PCV13, 13-valent pneumococcal conjugate vaccine.

After PCV13 introduction, meningitis incidence declined to 1.22 cases/100,000 person-years by 2015–16, a reduction of 70% (95% CI 54%–81%) from the pre-PCV7 period (Table 1; Figure 1). This decline was caused by the continuing reduction in PCV7-type disease and a large reduction in the additional PCV13 serotypes (1.56 cases/100,000 person-years [pre-PCV13] to 0.09 cases/100,000 person-years [2015–16]) while meningitis incidence caused by non-PCV13 serotypes remained static. Nearly all cases in 2015–16 were caused by non-PCV13 serotypes, and only 3 cases were caused by a PCV13 serotype (Table 1; Figure 5).

### Cases in Patients 5–64 Years of Age

Among patients 5–64 years of age, PCV7 serotypes were responsible for 46.2% (54/117), additional PCV13 for 18.8% (22/117), and non-PCV13 for 35.0% (41/117) of pneumococcal meningitis cases during the pre-PCV7 period. In this age group, meningitis incidence increased after PCV7 introduction, peaking in 2008–09 before declining after PCV13 introduction (Figure 1). After PCV7 introduction, PCV7-type meningitis declined, and additional PCV13-type meningitis increased but then declined after PCV13 introduction. Meningitis caused by non-PCV13 serotypes increased after PCV7 introduction and then stabilized during the PCV13 period. During 2015–16, the additional PCV13, especially serotype 3 (n = 5), caused some meningitis cases, but the non-PCV13 serotypes 12F (n = 10) and 8 (n = 9) were the predominant causes of meningitis; other serotypes caused only 1–2 cases.

### Cases in Patients >65 Years of Age

Before PCV7 introduction, the serotype distribution among patients >65 years of age was similar to that of patients 5–64 years of age, albeit with half the number of cases (Table 1). Meningitis incidence did not change after PCV7 introduction (0.60 cases/100,000 person-years [pre-PCV7] to 0.59 cases/100,000 person-years [pre-PCV13]) but declined substantially after PCV13 introduction to 0.27 cases/100,000 person-years by 2015–16, when serotypes 8 (25.0%, 6/24) and 23B (16.7%,4/24) predominated. In 2015–16, only 2 cases were caused by PCV13 serotypes (3 and 19A).

### Case-Fatality Rate

The overall CFR for pneumococcal meningitis was 17.5% (631/3,612; 95% CI 16.2%–18.7%) and pneumococcal nonmeningitis 19.9% (14,783/74,179; 95% CI 19.6%–20.2%). The CFR for pneumococcal meningitis was 10.7% (150/1,408; 95% CI 9.1%–12.4%) among patients <5 years of age, 17.3% (262/1,517; 95% CI 15.4%–19.3%) among patients 5–64 years of age, and 31.9% (219/686; 95% CI 31.8%–39.5%) among patients >65 years of age. The CFR for pneumococcal nonmeningitis was 3.5% (254/7,163) among patients <5 years of age, 10.8% (3,235/30,090) among patients 5–64 years of age, and 30.6% (11,292/36,907) among patients >65 years of age. The CFRs for patients with meningitis by serotype group were 14.2% (130/916) for PCV7 serotypes, 18.0% (143/793) for additional PCV13 serotypes, and 18.9% (290/1,534) for non-PCV13 serotypes.

In a logistic regression model, meningitis was associated with death (adjusted odds ratio [aOR] 1.6, 95% CI 1.4–1.7), independent of age group, serotype group, or period. Among meningitis cases, only increasing age (5–64 years aOR 1.7 [95% CI 1.3–2.1] and >65 years aOR 3.9 [95% CI 3.1–5.0] vs. children <5 years of age; p<0.0001 for both) was independently associated with death but not serotype group or surveillance year. In a logistic regression model comparing the CFR of serotypes associated with meningitis during the PCV13 period, only serotype 8 (CFR 33.7% [33/98] vs. CFR other serotypes 15.7% [123/783]) was associated with an increased odds of death (aOR 2.9, 95% CI 1.8–4.7; p<0.0001).

### Meningitis Cases Prevented

We estimated that 702 cases of meningitis were prevented during the 10 years since PCV7 introduction (2006–2016), mainly occurring after PCV13 introduction and nearly all in children <5 years of age. In total, 1,471 fewer cases were caused by PCV7 serotypes, and 173 more cases were caused by additional PCV13 serotypes (207 additional cases before PCV13 introduction and 34 fewer cases after PCV13 introduction).

## Discussion

In England and Wales, pneumococcal meningitis accounts for 5% of all IPD cases. Although large declines in IPD incidence were observed after PCV7 and PCV13 introduction, we observed a differential impact on pneumococcal meningitis and nonmeningitis. The annual incidence of pneumococcal meningitis remained unchanged after PCV7 introduction but declined by 48% after PCV13 replaced PCV7. The greatest decline in pneumococcal meningitis incidence (70%) was observed among children <5 years of age. By 2015–16, PCV13-serotype meningitis was rare, and nearly all cases were caused by non-PCV13 serotypes. The CFR, however, remained high (17.5%) and increased with age, but we found evidence of a lower CFR after both PCV7 and PCV13 implementation.

The reduction in PCV7-type pneumococcal meningitis after PCV7 was introduced in 2006 was rapidly offset by an increase in cases caused by non-PCV7 serotypes, especially 7F and 19A (later included in PCV13) and especially among adults (*21*). The replacement of PCV7 by PCV13 in 2010, however, led to large declines in pneumococcal meningitis cases, mainly because of an 84% reduction in cases caused by the additional serotypes included in PCV13 without an increase in cases caused by non-PCV13 serotypes. Similar trends have been reported in Israel, where meningitis incidence declined only after PCV13 introduction (*22*). In France, PCV7 implementation led to a rebound in incidence of pneumococcal meningitis, with a 2.2-fold increase among children, including a 6.5-fold increase among those <2 years of age (*23*), followed by a 44% decline after PCV13 introduction (*16*,*24*). In contrast, many countries with established PCV programs reported declines in pneumococcal meningitis after implementation of each vaccine (*18*,*25*–*31*). In the United States, PCV7 implementation was associated with declines in PCV7-type pneumococcal meningitis across all age groups, but the overall incidence of pneumococcal meningitis in adults did not change because disease with non-PCV7 serotypes increased (*32*). Possible explanations for the variable observations include differences in immunization doses and schedules, implementation of catch-up programs along with vaccine introduction, vaccine uptake rates, rapidity of increases in disease attributable to other serotypes after the introduction of each vaccine, and differences in the replacing serotypes’ abilities to cause IPD and meningitis.

We observed shifts in serotypes causing meningitis over time. Before PCV7 implementation, serotype 14 caused most meningitis cases (*10*), consistent with findings in Germany, Belgium, and Brazil (*33*–*35*). During the PCV7 period, serotypes 7F and 19A (*12*), among others, emerged as the most common replacing serotypes causing meningitis in Europe and the United States, mainly through clonal expansion (*36*,*37*). In several countries, including France and the United States but not the United Kingdom, isolates of the emerging serotype 19A exhibited high rates of resistance to multiple antimicrobial drugs (*38*,*39*). Meningitis caused by serotype 7F has been associated with more severe disease (increased complications, higher CFR) in children than meningitis caused by other serotypes (*40*).

The replacement of PCV7 with PCV13, which covers serotypes 7F and 19A, led to a rapid reduction in IPD, including meningitis, caused by these serotypes across all age groups. Marked reductions in IPD caused by these 2 serotypes were observed especially among children <2 years of age, the age group in which incidence of pneumococcal meningitis is highest (*12*). By 2015–16, serotypes 8 and 12F were the main replacing serotypes causing meningitis across all age groups.

The predominance of serotype 8 appears to be unique to the England and Wales population (*41*). In Germany, serotypes 14 and 6B pre-PCV7 and serotype 7F post-PCV7 were the most common; whereas in England and Wales, serotypes 12F, 8, and 10A caused nearly half of all pneumococcal meningitis cases in patients <5 years of age after PCV13 introduction (*25*). Of the most frequent serotypes isolated after PCV13 introduction in France (15B/C, 22F, 23B, 24F), only 24F had a high disease potential; 5 years after PCV13 introduction, no serotype predominated, and no significant increase in non–PCV13-type meningitis occurred (*42*). A common feature shared among countries with established PCV13 programs is the high proportion of cases (70%–80%) caused by non-PCV13 serotypes after PCV13 introduction (*20*,*43*).

In our cohort, the differences in disease trends among meningitis and nonmeningitis presentations after PCV7 and PCV13 implementation are probably a result of the different propensities of specific serotypes to cause meningitis, pneumonia, severe disease, and fatal outcomes. For example, before PCV7 introduction, some PCV7 (6B, 18C, 19F, 23F), additional PCV13 (3, 6A), and non-PCV13 (12F) serotypes were more likely to be associated with meningitis in the United Kingdom, while serotype 1 (additional PCV13) was less likely (*10*). Many of the serotypes that increased in incidence after PCV13 implementation did not exhibit a higher propensity for causing meningitis. Our findings (and those of others) highlight the importance of monitoring infectious diseases by their major clinical presentations. In the United States, after PCV7 introduction, the rates of meningitis and invasive pneumonia caused by non-PCV7 types increased for all age groups, whereas primary bacteremia rates did not change (*32*). In contrast, a study in Spain showed a shift in pneumococcal meningitis cases to persons in older age groups (*18*). In Canada, a higher proportion of IPD cases presented as meningitis after the introduction of PCV7 and PCV13 (*44*), and in Israel, disease caused by non-PCV13 serotypes increased by 256% for pneumococcal meningitis among children, 302% for pneumococcal bacteremic pneumonia, and 116% for other IPD presentations (*15*).

The CFR of our cohort (17.5%, 10.7% in children <5 years of age) was similar to that reported for the pre-PCV7 period (14.5%) but higher than that reported for children in Spain (5%) and the United States (8.3%–11.2%) (*2*,*18*,*31*). We have reported that nearly half of all children with IPD who died had meningitis (*4*) and that meningitis was an independent risk factor for death in children with IPD (*45*). Similar to our cohort, studies conducted in other countries have also indicated little change in CFR for pneumococcal meningitis after PCV introduction, despite significant reductions in disease incidence (*18*,*31*,*46*). Clinical follow-up of cases suggests that although the risk for pneumococcal meningitis was lowered after PCV introduction, once meningitis develops, the outcomes in terms of death or long-term sequelae are similar, irrespective of infecting serotype (*20*). Our finding of lower odds of meningitis but higher risk for death with serotype 8 meningitis is novel (*41*) and needs to be verified for other populations.

The strengths of our study included established, long-term national surveillance along with a national reference laboratory for IPD covering a large population of 55 million persons across England and Wales. A study limitation was that bacterial meningitis cases caused by unknown species types, a substantial proportion of which were probably *S. pneumoniae*, would not have been captured in the surveillance, thus leading to an underestimation of the burden of pneumococcal meningitis. In addition, lumbar punctures are less likely to be performed on adults than children, and therefore, some IPD cases in adults were probably not reported as meningitis. Another limitation was that enhanced surveillance with questionnaires for vaccine-eligible children only began when PCV7 was introduced. This change could potentially have led to improved ascertainment of meningitis cases in the vaccine-eligible cohort after PCV7 introduction and, therefore, caused the effects of PCV7 to be underestimated. On the other hand, cases in and disease trends among older children and adults should not have been affected because enhanced surveillance was restricted to children <5 years of age. Overall, however, these differences were unlikely to affect the trends over time, effect of vaccination, or serotype distribution among meningitis and nonmeningitis cases. Finally, some pneumococcal serotypes exhibit cyclical trends, which could potentially explain some of the observed changes in serotype distribution; we did not evaluate changes in individual serotypes over time because of the relatively small numbers of meningitis cases caused by individual serotypes.

Comparisons of our findings with those found in studies of other populations should be made cautiously because of differences in the distribution of serotypes causing invasive disease in persons in different age groups, propensities of individual serotypes causing meningitis, replacement serotypes causing disease after PCV7 and PCV13 introduction, secular trends in non-PCV13 serotypes, vaccination schedules and coverage, antimicrobial use, emergence of resistant serotypes, clinical practices for investigating and treating patients with suspected meningitis, surveillance methods, case definitions, and completeness of case ascertainment. Of note, in the United Kingdom, antimicrobial resistance among invasive pneumococcal isolates remains low (*47*,*48*).

In conclusion, the childhood pneumococcal vaccination program has reduced the incidence of IPD, including pneumococcal meningitis, across all age groups through a combination of direct and herd protection. We estimated that >700 cases of pneumococcal meningitis were prevented during the first decade of the program, although CFRs across different age groups remain relatively unchanged. By 2015–16, most cases of pneumococcal meningitis were caused by non-PCV13 serotypes. Further studies are needed to assess the risk factors, clinical course, and outcomes of pneumococcal meningitis associated with the replacing serotypes. Higher-valent vaccines are needed to target the emerging serotypes in the short term until serotype-independent vaccines become available.
